# Supplementary material for: People with severe mental illness have low rates of screening for non-communicable diseases: Findings of a multi-country cross-sectional study in South Asia
Source: Glob Ment Health (Camb). 2026 Feb 24;13:e45. doi: 10.1017/gmh.2026.10157 (PMC12973245; doi:10.1017/gmh.2026.10157)
Supplement: Appuhamy et al. supplementary material [file S2054425126101575sup001.docx]

**People with severe mental illness have low rates of screening for non-communicable diseases: Findings of a multi-country cross-sectional study in South Asia - Appendix**

***Participant Flowchart:***


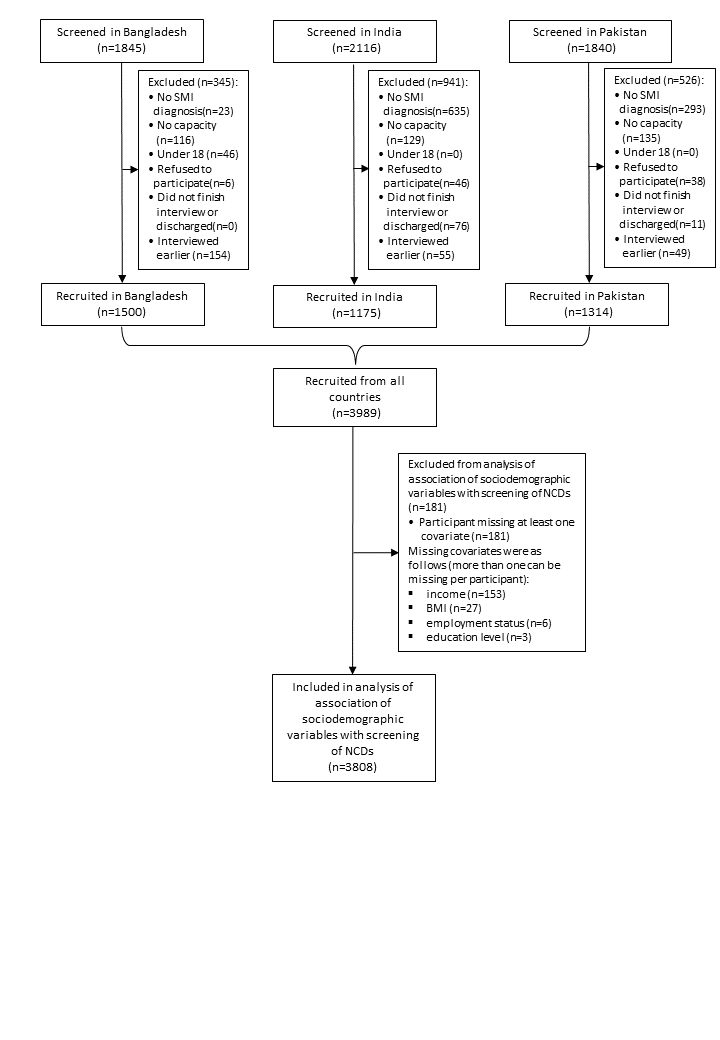


***Univariate model results:***

|  | **Self-reported screening of at least one NCD or NCD risk factor** | **OR (95% CI)** | **p-value** |
| --- | --- | --- | --- |
| **BMI Category** |  |  |  |
| Normal weight | 923/1760 (52.4) | Reference | p=. |
| Underweight | 125/298 (41.9) | 0.67 (0.53-0.86) | p=0.001 |
| Overweight | 695/1149 (60.5) | 1.39 (1.20-1.61) | p<0.001 |
| Obesity | 406/601 (67.6) | 1.78 (1.47-2.14) | p<0.001 |
| **Sex** |  |  |  |
| Male | 1225/2283 (53.7) | Reference | p=. |
| Female | 924/1525 (60.6) | 1.27 (1.11-1.44) | p<0.001 |
| **Age group** |  |  |  |
| 18-24 years | 307/695 (44.2) | Reference | p=. |
| 25-39 years | 969/1797 (53.9) | 1.44 (1.21-1.71) | p<0.001 |
| 40-54 years | 636/988 (64.4) | 2.19 (1.81-2.66) | p<0.001 |
| 55+ years | 237/328 (72.3) | 3.07 (2.34-4.04) | p<0.001 |
| **SMI diagnosis** |  |  |  |
| Bipolar disorder | 827/1393 (59.4) | Reference | p=. |
| Major depressive disorder   with psychotic features | 455/720 (63.2) | 1.20 (1.00-1.44) | p=0.053 |
| Schizophrenia-type disorder | 867/1695 (51.2) | 0.72 (0.63-0.83) | p<0.001 |
| **Setting** |  |  |  |
| Inpatient | 350/662 (52.9) | Reference | p=. |
| Outpatient | 1799/3146 (57.2) | 1.07 (0.91-1.26) | p=0.402 |
| **Level of education** |  |  |  |
| No formal education | 308/518 (59.5) | Reference | p=. |
| Primary education | 725/1422 (51.0) | 0.73 (0.60-0.89) | p=0.002 |
| Secondary education | 392/699 (56.1) | 0.87 (0.70-1.09) | p=0.241 |
| Higher/more than    secondary | 724/1169 (61.9) | 1.14 (0.93-1.40) | p=0.222 |
| **Work status**  **(past 12 months)** |  |  |  |
| Employed | 806/1424 (56.6) | Reference | p=. |
| Unemployed | 534/1064 (50.2) | 0.79 (0.68-0.93) | p=0.004 |
| Other^1^ | 809/1320 (61.3) | 1.17 (1.00-1.35) | p=0.043 |
| **Income tertile** |  |  |  |
| Low | 853/1573 (54.2) | Reference | p=. |
| Middle | 678/1254 (54.1) | 0.99 (0.85-1.14) | p=0.848 |
| High | 618/981 (63.0) | 1.43 (1.22-1.69) | p<0.001 |
| **Country** |  |  |  |
| Bangladesh | 822/1494 (55.0) | Reference | p=. |
| India | 501/1023 (49.0) | 0.76 (0.65-0.89) | p<0.001 |
| Pakistan | 826/1291 (64.0) | 1.47 (1.26-1.71) | p<0.001 |

***Direction and effect sizes of significant covariate interactions with country***

Additional individual models were fitted including an interaction term between country and each variable. When comparing to the model with no interactions, the likelihood ratio test identified variables sex (p=0.003), patient setting (p=0.0496), SMI diagnosis (p<0.001), work status (p=0.001) and income (p=0.023) as having a significant improvement to the fit of the model when an interaction effect with country is included. The direction and effect of these interaction terms can be found in the Appendix.

| **Covariate interaction term with country (Reference: Bangladesh)** | **OR (95% CI)** | **p-value** |
| --- | --- | --- |
| **Sex (Reference: Male)** |  |  |
| Female & India | 0.71 (0.51-0.99) | p=0.044 |
| Female & Pakistan | 1.32 (0.94-1.85) | p=0.111 |
| **Setting (Reference: Inpatient)** |  |  |
| Outpatient & India | 1.34 (0.89-2.01) | p=0.156 |
| Outpatient & Pakistan | 0.70 (0.42-1.16) | p=0.164 |
| **SMI Diagnosis (Reference: Bipolar disorder)** |  |  |
| Major depressive disorder with psychotic features & India | 0.32 (0.14-0.72) | p=0.006 |
| Major depressive disorder with psychotic features & Pakistan | 0.71 (0.39-1.28) | p=0.251 |
| Schizophrenia-type disorder & India | 0.80 (0.57-1.14) | p=0.225 |
| Schizophrenia-type disorder & Pakistan | 0.46 (0.30-0.71) | p<0.001 |
| **Employment Status (Reference: Employed)** |  |  |
| Unemployed & India | 2.45 (1.58-3.82) | p<0.001 |
| Unemployed & Pakistan | 1.26 (0.84-1.89) | p=0.257 |
| Other (student, homemaker, retired) & India | 1.21 (0.82-1.80) | p=0.334 |
| Other (student, homemaker, retired) & Pakistan | 1.29 (0.87-1.90) | p=0.205 |
| **Income tertile (Reference: Low)** |  |  |
| Middle & India | 0.69 (0.47-1.01) | p=0.054 |
| Middle & Pakistan | 0.69 (0.47-1.01) | p=0.057 |
| High & India | 0.50 (0.32-0.79) | p=0.003 |
| High & Pakistan | 0.73 (0.49-1.08) | p=0.119 |
